# An online intervention for carers to manage behavioral symptoms in motor neuron disease (MiNDToolkit): a randomized parallel multi-center feasibility trial

**DOI:** 10.1080/21678421.2024.2350658

**Published:** 2024-05-15

**Authors:** E. Mioshi, K. Grant, E. Flanagan, S. Heal, H. Copsey, R.L. Gould, M. Hammond, L. Shepstone, P.A. Ashford

**Affiliations:** 1 School of Health Sciences, University of East Anglia, Norwich, UK; 2 Norwich Clinical Trials Unit, Norwich Medical School, University of East Anglia, Norwich, UK; 3 MND Association Norfolk, Norwich & Waveney Branch, Norwich, UK; 4 Norfolk MND Care and Research Network, Norwich and Norfolk University Hospitals, Norwich, UK, and; 5 Division of Psychiatry, University College London, London, UK

**Keywords:** Amyotrophic lateral sclerosis, motor neurone disease, carer, caregiver, trial, behavioral symptoms, ALSFTD, feasibility

## Abstract

**Background:**

Evidence on management of behavioral symptoms in motor neuron disease (MND) is lacking. The MiNDToolkit, an online psychoeducational platform, supports carers dealing with behavioral symptoms (BehSymp). The study objectives were to ascertain recruitment and retention rates, carer and healthcare professional (HCP) use of the platform, and completion of online assessments, to inform a full-scale trial. *Design*: Randomized, parallel, multi-center, feasibility trial.

**Setting:**

England and Wales, across diverse MND services; recruitment from July/21 to November/22; last participant follow-up in March/23.

**Participants:**

Carers of people with motor neuron disease (PwMND) with BehSymp, recruited through MND services. After confirming eligibility, participants completed screening and baseline assessments online via the MiNDToolkit platform and were randomized centrally in a 1:1 ratio to MiNDToolkit or control.

**Intervention:**

MiNDToolkit offered tailored modules to carers for the 3-month study period. Carers in the intervention group could receive additional support from MiNDToolkit trained HCPs. The control group was offered access to the intervention at the end of the study. Data were collected on platform usage and psychosocial variables.

**Main outcomes:**

One hundred and fifty-one carers from 11 sites were invited to join the study (letter, face-to-face); 30 were screened; 29 were randomized. Fifteen people were allocated to the control arm; 14 to intervention. Carers were mostly female; median age for was 62.5 (IQR: 58, 68; intervention) and 57 (IQR: 56, 70; controls). Study retention was high (24/29 = 82.76%); carers engaged with the platform on average 14 times (median (IQR):14.0 (10.0, 18.5)) during the study period.

**Conclusion:**

The MiNDToolkit study was feasible and well accepted by carers and trained HCPs. A definitive trial is warranted.

## Introduction

Around 50–75% of people with motor neuron disease (PwMND) present with progressive (1) behavioral symptoms (BehSymp) commonly seen in frontotemporal dementia (FTD) such as apathy, disinhibition, rigidity (2), and deficits in social cognition (3). International consensus criteria on the diagnosis of motor neuron disease (MND) with FTD-like symptoms have been published (4) to support clinical identification, management, and research studies.

Behavioral symptoms can negatively affect clinical decision making, which are particularly pressing in MND given the rapid progressive changes in mobility, swallowing, and breathing (5). Additionally, PwMND with severe apathy and disinhibition (6,7), or those with MNDFTD (8) have worse prognosis. Finally, cognitive and BehSymp have been associated with greater carer burden and distress in spouses (9,10), children (11), and healthcare professionals (HCPs) (12).

The National Institute for Health and Care Excellence (NICE, London, UK) guidelines for the management of MND highlight the importance of professionals’ recognition and assessment of these symptoms (13), but, despite the negative impact of such symptoms in the prognosis of MND, and the wellbeing of their carers, there is marked paucity of evidence on the clinical management of behavioral and cognitive symptoms in MND.

No research study testing an intervention to support carers in managing BehSymp in MND has been identified. This study aimed to assess the feasibility of conducting a multi-center, randomized controlled trial of MiNDToolkit, a novel online intervention to support carers in the management of BehSymp.

## Methods

### Design

A multi-site, two-arm, parallel, randomized, controlled feasibility trial was conducted, allocating carers of people with MND, in a 1:1 ratio to a “usual care” arm or an intervention arm who were given access to the MiNDToolkit online platform for three months. After three months, all participants completed follow-up questionnaires and the intervention was made available to the control participants, with all participants offered continued access to the platform until the end of the trial. A nested qualitative process evaluation, including HCP acceptability of MiNDToolkit, is described separately (14). Ethical approval: London Queen Square Research Ethics Committee (19/LO/0692, IRAS260290).

### Participants

Potential participants were identified and referred by clinical teams. Inclusion criteria were: family or live-in professional carers of PwMND with BehSymp, or MNDFTD (4); aged ≥18 years; having ≥7 hours of contact with the PwMND/week; able to communicate in English without support; not a carer of a PwMND who already has a carer recruited into the study. Eligibility was checked in two stages: sites identified carers fulfilling the above demographic characteristics, and the online platform screened for symptoms via online questionnaires.

### Sample size

A sample size of 20–30 participants was chosen following published recommendations for feasibility trials (15,16). It was considered that this would be a sufficient sample to report on the practicalities of delivering the novel online intervention, recruitment, uptake, and attrition.

### Procedures

Clinical teams were asked to consider all families affected by MND in their caseload to identify potentially eligible carers. Carers attending routine appointments were informed about the study (in-person, telephone, and post). Carers consented to share contact details with the research team, with staff completing a consent to contact form on behalf of the potential participant. All study information was co-designed with carers from the carer-research involvement group.

Upon receipt of a referral, a researcher telephoned potential participants, and sent an information sheet via email. If the participant confirmed interest in the study, they were issued a login for the MiNDToolkit platform, allowing them to provide an electronic informed consent and clinical screening to confirm eligibility. The platform algorithm behind the user-friendly interface calculates assessments automatically, so a participant was then taken to the trial activities or diverted to a “thank you” message if they did not pass screening. Additional information is given in the Appendix.

## Randomization

A randomization list created by the Norwich CTU, using random blocks of 2 and 4, with equal allocation, i.e. a ratio of 1:1 for intervention to control was used. Allocation was concealed prior to randomization; the list was held only by members of the data management team. Due to the nature of the psychoeducational intervention, blinding of allocation was not possible.

## Intervention: MiNDToolkit

MiNDToolkit is a psychoeducational intervention delivered via a bespoke online platform. HCP reinforcement of the intervention during routine clinical contacts is recommended, with MiNDToolkit training (14) provided to professionals involved at trial sites.

MiNDToolkit contains 16 modules, comprising BehSymp in MND and strategies to deal with symptoms (Table 1). Following advice from carers, modules are short and time to complete is shown before modules are selected. Animations are simple and audio content is prioritized, e.g. video clips, simple animations. The online platform is the result of an extensive adaptation process of the original material in 2020 (17), transforming the paper-based to an online-based intervention, ensuring that MiNDToolkit could be used even during lockdowns.

**Table 1.** MiNDToolkit modules offered to carers.

Compulsory modules	Modules on symptoms	Modules on strategies
What is MND? How common are non-motor changes?	What is apathy?	Encourage and prompt
Which are the brain changes in MND?	What is disinhibition?	Adapt and accommodate
Top tips from carers, to carers	What is rigidity?	Simplify and clarify
Looking after your wellbeing	What are deficits in social cognition?	Prepare and increase awareness
	What are hallucinations?	Support and share decisions
	What is lack of insight into own changes?	
	What are eating changes?	

Compulsory modules were presented at the beginning and end of the menu to all. Modules on symptoms and strategies were tailored to carers’ questionnaires responses or were made available by the HCP.

**Table 2.** MiNDToolkit feasibility trial outcomes (estimated proportions and 95% exact confidence intervals).

Measure^a^	Proportion	Exact^b^ lower 95% confidence interval	Exact^b^ upper 95% confidence interval
Approached rate (approached/screened)	151/284 0.5317	0.4718	0.5909
Recruitment rate (consented/approached)	30/151 0.1987	0.1382	0.2713
Randomization rate (randomized/consented)	29/30 0.9667	0.8278	0.9992
Attrition rate (to end of FU) (withdrawals/randomized)	1/29 0.0345	0.0009	0.1776
Reaching FU rate	24/29 0.8276	0.6423	0.9415
FU questionnaires abandoning	1/24 0.0417	0.0011	0.2112
FU completion outside 30 days	2/23 0.0870	0.0107	0.2804

^a^FU: follow-up after 3 months.

^b^Exact 95% confidence interval used (Clopper–Pearson method) due to small sample size and proportions often close to 0 or 1.

**Table 3.** MiNDToolkit feasibility trial: carer descriptive statistics at baseline and follow-up.

	Intervention group		Control group	
	Baseline (*n* = 14)	Follow-up (*n* = 11)	Baseline (*n* = 15)	Follow-up (*n* = 13)
Age of carer: median (IQR)	62.5 (58.0, 68.0)	64.0 (58.0, 69.0)	57.0 (55.0, 70.0)	60.0 (56.0, 70.0)
Gender of carer, female: n (%)	11 (78.6%)	9 (81.8%)	12 (80.0%)	10 (76.9%)
Ethnicity of carer: *n* (%)				
Black/African	1 (7.1%)	1 (9.1%)	1 (6.7%)	0
Caucasian	10 (71.4%)	9 (81.8%)	12 (80.0%)	11 (84.6%)
Other	3 (21.4%)	0	1 (6.7%)	2 (15.4%)
Prefer not to say	0	1 (9.1%)	1 (6.7%)	0
Relationship to person with MND: *n* (%)				
Parent	1 (7.1%)	1 (9.0%)	0	0
Son/daughter	1 (7.1%)	1 (9.0%)	3 (20.0%)	1 (7.7%)
Spouse/partner	12 (85.7%)	9 (81.8%)	12 (80.0%)	12 (92.3%)
Live in same household: *n* (%)				
Yes	12 (85.7%)	9 (81.8%)	14 (93.3%)	12 (92.3%)
Carer employment status: *n* (%)				
Full-time	5 (35.7%)	3 (27.3%)	3 (20.0%)	3 (23.1%)
Part-time	2 (14.3%)	1 (9.0%)	4 (26.7%)	2 (15.4%)
Not working	0	1 (9.0%)	1 (6.7%)	1 (7.7%)
Retired	7 (50.0%)	6 (54.6%)	7 (46.7%)	7 (53.9%)
Reduced work to care: *n* (%)				
Yes	5 (35.7%)	3 (27.3%)	4 (26.7%)	5 (38.5%)
Number of months as carer: median (IQR)	15.0 (7.0, 24.0)	20.0 (12.0, 24.0)	18.0 (12.0, 40.0)	35.0 (16.0, 48.0)
Carer education level: *n* (%)				
PhD	0	0	1 (6.7%)	2 (15.4%)
Master’s degree	1 (7.1%)	1 (9.1%)	2 (13.3%)	2 (15.4%)
Bachelor’s degree	6 (42.9%)	4 (36.4%)	3 (20.0%)	1 (7.7%)
Secondary school: A level	2 (14.3%)	2 (18.2%)	2 (13.3%)	1 (7.7%)
Vocational	4 (28.6%)	4 (36.4%)	3 (20.0%)	3 (23.1%)
Secondary school: O level/GCSE	1 (7.1%)	0	3 (20.0%)	3 (23.1%)
Primary school	0	0	1 (6.7%)	1 (7.7%)

The MiNDToolkit platform is adaptive. Modules offered, defined by an algorithm, vary per participant at each assessment point. Modules match BehSymp reported, e.g. if the PwMND has few symptoms, few modules are shown. Carers are asked to complete two modules/week and are informed that the access to the platform will cease after three months. Automatic reminders for module completion are emailed weekly, and carers can opt-in to receive a SMS reminder. Carers can, however, choose to complete all modules at once if they wish. There is no restriction as to how long they can access the modules per week, and they can replay modules during the 3-month period, based on carer consultation *a priori*.

A minimum of two HCPs from each site were trained to reinforce the content of the MiNDToolkit at every opportunity, e.g. clinical appointment, phone call, home visit. HCPs were asked to record their points of contact on MiNDToolkit, if they were able to reinforce the content, and what the result was of that discussion, including if they did not have time to reinforce MiNDToolkit content.

The decision to move from primary HCP delivery (paper, pre-pandemic) to HCP reinforcement (online MiNDToolkit) was a deliberated response. The goal was to facilitate clinical teams’ engagement with MiNDToolkit, while ensuring carers could have access to research studies regardless of COVID-19 lockdowns—also potentially facilitating implementation and national scaling-up of MiNDToolkit in future.

## Control: treatment as usual

Current MND specialist care does not entail standardized provision of care for BehSymp management. Some HCPs may advise if they have clinical experience, but no clear approaches are recognized. This lack of standardized provision and training has been further explored in the process evaluation (14). As such, carers in the control group were effectively a waiting list control group.

## Data collection

At time of referral, sites checked their caseloads and reported if the carer was eligible; reasons if not eligible; if invitation was in person or by letter; reason for not approaching; reason for declining. Quantitative data from participants were collected via MiNDToolkit. Screening and baseline assessments were undertaken after consent was obtained, prior to randomization.

Screening measures included: carer socio-demographics, ALS-Functional Rating Scale Revised (18) (ALSFRS-R) and Motor Neurone Disease Behavioral Instrument (MiND-B) (2).

The screening assessment was automatically scored by MiNDToolkit, and carers meeting the eligibility criteria were seamlessly taken to baseline data collection. If the criteria were not met, the carer was invited three months later for a repeat screening assessment, when symptoms of the PwMND may have changed. Participants were followed-up at three months after randomization.

## Primary outcome measures

Feasibility outcomes were collected to enable an estimation of key parameters to inform a future trial, and to provide preliminary information about the impact of the intervention:

1. *Recruitment feasibility, randomization acceptability*: Number of potential participants assessed for eligibility (including reasons for exclusion/non-participation), number consented to be randomized.
2. *Intervention acceptability and fidelity (carers and HCPs):* By qualitative interviews; study attrition in the intervention phase, use of the MiNDToolkit, in particular engagement with MiNDToolkit: number of times accessed; length of time spent logged in; modules repeated (see (14,19)).
3. *Completeness of outcome measures:* Number of non-completed outcomes, time to completion.

## Secondary outcome measures

1. *Efficacy outcomes:* Variables related to the carer were collected within one month of the end of the intervention: depressive symptoms (PHQ-9) (20); anxiety (GAD-7 (21)); quality of life (CES (22)), and wellbeing from a capability perspective (ICECAP-A (23)).

## Analysis

Feasibility outcomes were reported descriptively and narratively. The analysis of efficacy outcome measures was descriptive, reported as medians and interquartile ranges (IQRs) and numbers and percentages for binary and categorical variables. Descriptive statistics on MiNDToolkit engagement were reported. Formal analyses were conducted in terms of efficacy for questionnaire outcomes using generalized linear models (GLMs). However, these are for information purposes only, as efficacy is not our focus for this study. HCP and carer acceptability are reported separately (14,19).

## Trial monitoring and safety

A Trial Management Group assisted with developing the design, co-ordination, and strategic management of the trial, overseeing safety. Carers could report adverse events at follow-up assessment. Researchers and linked HCPs were automatically notified if a carer indicated that they were experiencing thoughts of self-harm (PHQ-9), and a list of contact details for support and crisis lines would appear on the platform for the carer.

## Results—primary analyses

### Recruitment and retention

Two hundred and eighty-four MND families were assessed for eligibility, with 151 carers invited to the study via letter or face-to-face. Of these, 30 were screened, and 29 passed the clinical screening in their first attempt and were randomized, 14 to the intervention and 15 to the treatment as usual group.

**Figure 1.**
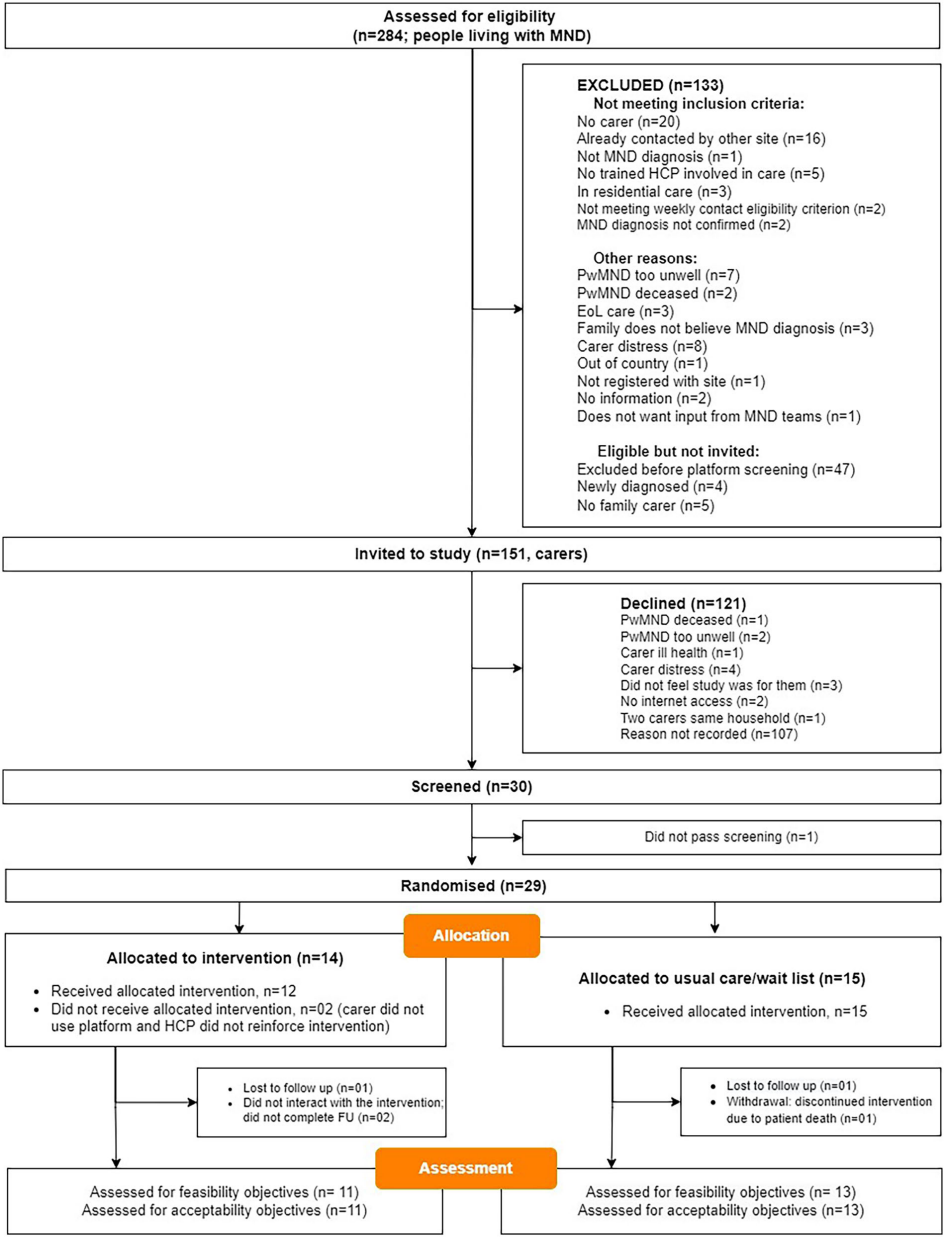
MiNDToolkit feasibility trial CONSORT flow diagram for randomized feasibility trials.

**Figure 2.**
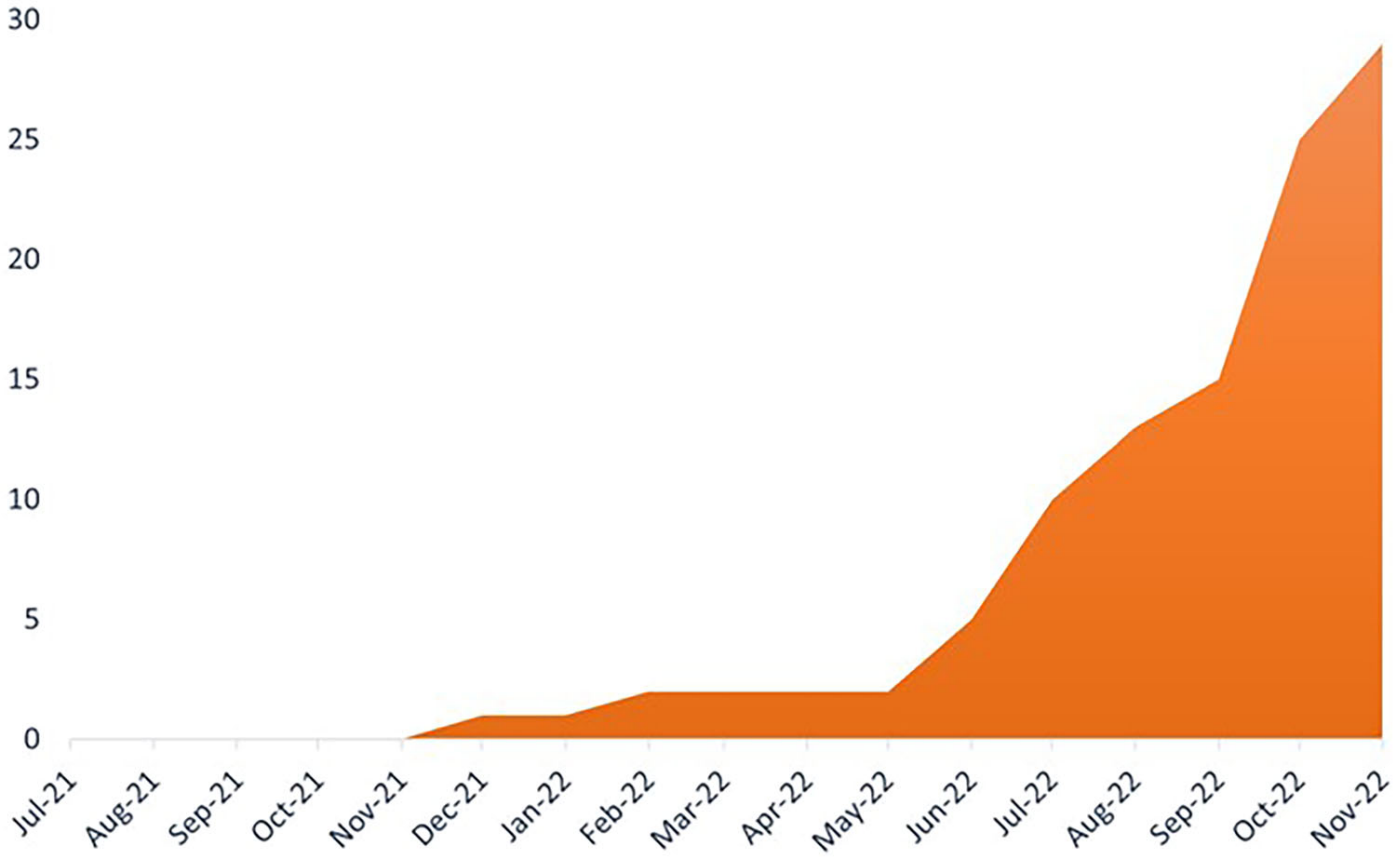
MiNDToolkit feasibility trial recruitment progression from first site opening for recruitment in July/2021, to last (11th) site starting site recruitment in November/2022.

Reasons for exclusion and number of people eligible but not invited are shown in Figure 1. Follow-up occurred between March/22 to March/23, with the trial ending when the last participant completed their 3-month follow-up assessments two participants were lost to follow-up (one per arm), two participants did not start the intervention or complete follow-up assessment (Figure 2). Carer attrition rate due to PwMND decline/death was much lower than anticipated, with only one carer discontinuing the study before the follow-up assessment. Overall, 24 participants were assessed for the feasibility and acceptability objectives (Table 2).

### Participant characteristics

Carers were mostly female in both groups. Median age was 62.5 for carers in the intervention group, and 57 for carers in the control arm. Ethnicity was mixed, with 71.4% Caucasian, and remaining categorized as Other or Black. Approximately, 50% of carers were engaged in paid work, part- or full-time; around a quarter of all carers reduced work hours to provide care for the person with MND (Table 3). Demographic characteristics of PwMND are shown in eTable 1.

### MiNDToolkit—carer use

From those allocated to intervention, 12/14 carers interacted with the intervention modules, on average, 14 times (median (IQR): 14.0 (10.0, 18.5)) during the 3-month intervention period. Length of engagement per interaction on MiNDToolkit was measurable, since MiNDToolkit was pre-set to log out after 60 minutes, and people usually do not log out when moving to another browser or activity, leading to inflated values of engagement. For this reason, we are not reporting the length of engagement per interaction.

### MiNDToolkit—completion of outcome measures

Outcome measures of participants were completed in full, without accidental missed items. Questionnaire items cannot be missed because the platform was set up to ensure participants would only move forward after entering a response, with pre-set parameters, e.g. selecting a response for multiple choice questions, or for example, entering a digit if the question was open and referring to months. The median time taken to complete baseline questionnaires was 36 minutes (median = 36, IQR = (21.0, 153.0), range = (6.0, 38965.0)), compared to 28 minutes at follow-up (median = 28.0, IQR = (19.0, 52.0), range = (10.6, 45010.0)), with some participants completing the full set of questionnaires in one session, while others completed this over several days. Note that the platform calculated the time between the sessions, over days, not per session.

## Results—efficacy outcomes

Median scores suggested mild anxiety for the intervention (median = 7; IQR = 4, 8) and control (median = 5; IQR = 2, 7) groups at baseline. Depressive symptoms were low for the intervention (median = 7; IQR = 3, 10) and control (median = 4; IQR = 3, 8) groups; scores ≤4 reflect absence of depressive symptoms. However, some carers reported clinically significant symptoms of depression and anxiety (eTable 2). Quality of life was low overall at baseline (median = 11; IQR = 9, 13) but capability was relatively high (median = 0.76; IQR = 0.61, 0.89).

Regarding the PwMND, MNDFRS-R scores were similar in both groups and reflected moderate-severe disability (overall 27/48 at baseline). Behavioral symptoms were prominent in both groups (Table 2).

We performed GLMs to formally test for a between group difference but found no evidence of a difference between groups for any of the questionnaire outcomes (eTable 3). The study was powered for feasibility and not efficacy; hence, caution is required to interpret effect estimates.

## Discussion

### Site recruitment

All sites referred recruited participants. Ten were National Health Service (NHS) UK sites and one charitable service was funded by the NHS to provide MND care. In the UK, NHS services are funded through central government via taxes, translating in free care at the point of access; as such, all PwMND for whom the participant were caring for, were receiving free care. Sites varied: five were recognized specialist MND Care Centers, one offered palliative care services, two were community-based and three services were from secondary hospitals. This diversity meant different frequencies of contact, types of care provided, and team composition. For example, specialist centers usually review families 3-monthly and include a neurologist or palliative care consultant, while community-based teams may have weekly contact, via a team of allied health professionals and nurses. Site principal investigators had varied professional backgrounds, with most being new to research.

Securing 11/24 sites took longer than intended. Three sites fell through, one for lack of communication after initial discussions, and two at the late stages of set up: team ill health, IT policy incompatibility. Another 10 sites expressed interest but were unable to deliver the study for various reasons, e.g. capacity issues despite team’s interest. Administrative delays in study set up also contributed to slow recruitment progress, indicating that early identification of or reconnection with previous sites from the feasibility study would be helpful in a future trial.

### Carer recruitment

151/284 families of PwMND identified as potential participants were invited to be screened for MiNDToolkit. Site initial screening was inconsistently reported, but the proportion of recruited carers from those invited (19.87%) indicates that recruitment to a larger trial would be feasible. Unfortunately, many reasons for non-response are unknown.

Protocol and procedural changes were implemented to facilitate recruitment and encourage HCP/site engagement:

- *Eligibility*: Reduction on number of contact hours with PwMND (from ≥14 to 7 hours/week), after carer feedback, recognizing that individuals could be the primary carer but not have many hours of contact.
- Introduction of mail invitations to enable participants to self-refer to the study, on site feedback.
- HCPs were sent thank you cards and care packs (e.g. chocolate) to celebrate achievement of recruitment target or over-recruitment.
- Monthly newsletters highlighted sites that performed well, acknowledging their performance and engagement.

Letters of invitation sent directly to potential participants not only facilitated study recruitment; it also demonstrated to HCPs that PwMND with BehSymp could be missed if not assessed. The gap between HCPs’ knowledge of, and experience in managing BehSymp, was also identified in the MiNDToolkit process evaluation (14). Uncertainty of how to deal with families’ response to the study may have initially created a barrier to study referral, which was likely overcome by the carers’ positive response to the feasibility trial mail out.

*Carer retention* rate was excellent compared to the few other studies focusing on MND carers, where almost 50% of carers dropped out (24), while in our study, 82.76% completed follow-up measures.

*Potential outcome measures for future studies* were explored in this study: carer quality of life, depressive symptoms, and anxiety. These are psychological wellbeing domains often evaluated in studies involving carers of PwMND (9,25), but unlikely candidates for future trial as median scores were mild for anxiety and non-clinical range for depression. There is evidence in other contexts that psychoeducational interventions can reduce anxiety symptoms in carers (26,27), but this is not a universal finding (28). Quality of life may not respond well to the intervention, as the potential benefits of MiNDToolkit may be tempered by the uncertain trajectory yet rapid progression of MND. Carers face numerous losses in rapid succession in MND, and their wellbeing will be affected by multiple factors. Outcomes relating to carer competence or skills, are more likely to respond to a psychoeducational intervention as shown in other carer trials in stroke (29) and dementia (30). Indeed, feedback from carers through the process evaluation (14) revealed that learning about BehSymp, and perceiving an ability to make changes in daily management was empowering and supported acceptance of these non-motor symptoms.

### Strengths and limitations

Strengths include high engagement of participants in MiNDToolkit usage and study completion, compared to another trial involving carers of PwMND (31), and echoes findings of carer demand for individual support (32). Our carers were diverse regarding ethnicity and employment status. Statistics showed that carers accessed MiNDToolkit early or late in the day; the online nature of MiNDToolkit made it accessible for carers who spend many hours providing care. Additionally, carer feedback in interviews (14) confirmed that MiNDToolkit was easy to use and engaging.

We identified simple modifications that can be made to the platform and training, e.g. additional video to demonstrate HCP role play, and paper documents as memory aides for the HCPs (14).

Limitations include small sample size and lack of blinding. One site was randomly allocated only carers in the control arm, thus limiting their ability to reinforce the intervention, which future per site randomization approaches would rectify. Reinforcement from the HCPs was variable and intended to be higher (14). This could be addressed via changes in the training and materials provided to increase clarity of the HCP role in the intervention, or increasing hours dedicated to the trial. As it was not possible to report the length of engagement per platform interaction, we were unable to examine variables that may have influenced this.

## Conclusion

To the best of our knowledge, the MiNDToolkit is the first multi-center RCT to investigate the feasibility of testing an online intervention for carers dealing with BehSymp in MND. Currently, no guidelines for the management of BehSymp in MND exist. Carers’ need of specialist support cannot be underestimated. MiNDToolkit is an acceptable, feasible psychoeducational intervention that addresses the needs of carers—and HCPs, to learn, understand and successfully manage these symptoms. A definitive RCT is warranted.

## Supplementary Material

Supplemental Material

## Appendix

### Additional information on site identification, retention, and engagement – MiNDToolkit feasibility study

We employed several strategies to promote the research study to teams involved in the provision of specialist MND care to those affected by MND in England and Wales. Strategies fell within three main categories: (1) contacting MND care centers, via email to all UK MND Care Centres listed on the MND Association website; (2) presenting the study at interest groups, e.g., MND Clinical Interest group and carers’ meetings; (3) reaching out to HCPs involved in MND care, e.g., reaching out to all HCPs who were members of the MND Association Community of Practice, organized by the MND Association, e.g., newsletter and via a video clip explaining the study and asking interested HCPs to get in touch. These actions were done consecutively, which helped recruitment over time. In future, however, we aim to use the same strategies as soon as funding is confirmed, in parallel, and continue promoting the full trial monthly from funding award, until recruitment ends.

Approximately 15 different teams approached the research team in total, demonstrating great interest in becoming a site involved in the feasibility study. Upon contact, emails were exchanged, and meetings were set up. A few sites were unable to take part once they learned of the requirements of the study; other teams/sites did not reply to the research team emails, despite initial interest. Length of time between initial contact and site set up was relatively long, taking more than 6 months in several instances.

Reasons for not confirming capability of a site included: lack of time capacity from the clinical team; site not able to access our online documentation due to IT set up; lack of research capability in a small hospice; PI illness; site management not supporting adoption of the study.

MND teams who became study sites were varied, ranging from specialist MND Care Centres to hospices. Team composition varied markedly and no pattern could be identified in a small feasibility study. HCP interest in the intervention was diverse, and professional background was also varied. Trained HCPs were asked to reinforce MiNDToolkit learning and strategies during appointments. Training for HCPs include a two-hour online session, a 90-min group training session with role playing, and optional weekly supervisions throughout the trial. In total, 28 HCPs were involved in the feasibility study: eight nurses, six occupational therapists, one neuropsychologist, one research practitioner, three speech and language therapists, four palliative care consultants, one neurology consultant, two physiotherapists, one dietitian, and one MND Healthcare Support Worker; five of them were MND Care Coordinators.

Screening logs were provided to all sites, alongside training in the Site Initiation Visit. Some sites had less experience in research, with many new Principal Investigators. In the UK, national governance requires basic training in clinical research, which was completed by some PIs while site was being set-up. Some screening logs were returned incomplete at the end of the study, despite numerous attempts in obtaining a final record for all sites. Many screening logs did not report on the reasons for carers not responding or declining the study.

### Recommendations for trials targeting recruitment of carers

For trials involving carers, some mitigation strategies are proposed, based on the learning from this feasibility study:

- Contacting potential sites before funding is secured. Our database currently has 24 potential sites for a future fully powered clinical trial.
- Start site set-up contracting a year before the sites are due to receive green light.
- Approach several sites concomitantly.
- Create mini video clips to support HCPs in further understanding which actions are research processes, and which actions are parts of the MiNDToolkit intervention per se.
- Provide specific training and associated visual materials for completion of the screening log.
