# Supplementary material for: An online intervention for carers to manage behavioral symptoms in motor neuron disease (MiNDToolkit): a randomized parallel multi-center feasibility trial
Source: Amyotroph Lateral Scler Frontotemporal Degener. 2024 May 15;25(5-6):506–16. doi: 10.1080/21678421.2024.2350658 (PMC11286211; doi:10.1080/21678421.2024.2350658)
Supplement: Supplemental Material [file IAFD_A_2350658_SM3187.docx]

**Supplement: Online-only material**

**eTable 1:** Baseline Descriptive Statistics of people with MND who were supported by the carer in the MiNDToolkit Study

|  | Intervention group (n=14) | Control group (n=15) | Overall (n=29) |
| --- | --- | --- | --- |
|  |  |  |  |
| Age of person with MND: median (IQR) | 65 (61, 72) | 67 (59, 75) | 66 (61, 72) |
|  |  |  |  |
| Gender, male: n (%) | 10 (71.4%)s | 10 (66.7%) | 20 (69.0%) |
|  |  |  |  |
| MND diagnosis as reported by carer: n (%)  ALS  Bulbar onset MND/Progressive bulbar palsy  Dementia  Primary lateral sclerosis  Progressive muscular atrophy  Not sure | 6 (42.9%)  2 (14.3%)  3 (21.4%)  3 (21.4%)  0  2 (14.3%) | 6 (40.0%)  4 (26.7%)  0  1 ( 6.7%)  1 ( 6.7%)  4 (26.7%) | 12 (41.4%)  6 (20.7%)  3 (10.3%)  4 (13.8%)  1 ( 3.5%)  6 (20.7%) |
|  |  |  |  |
| Employment status: n (%)  Full-time  Part-time  Not working  Retired | 0  1 ( 7.1%)  3 (21.4%)  10 (71.4%) | 1 ( 6.7%)  0  4 (26.7%)  10 (66.7%) | 1 ( 3.5%)  1 ( 3.5%)  7 (24.1%)  20 (69.0%) |
|  |  |  |  |

**eTable 2.** MiNDToolkit feasibility trial: Carer Questionnaire outcome descriptive statistics at Baseline (n=29) and Follow-up (n=24)

|  | Intervention group | | Control group | | Overall | |
| --- | --- | --- | --- | --- | --- | --- |
|  | **Baseline**  **(n=14)** | **Follow-up**  **(n=11)** | **Baseline**  **(n=15)** | **Follow-up**  **(n=13)** | **Baseline**  **(n=29)** | **Follow-up**  **(n=24)** |
|  |  |  |  |  |  |  |
| GAD total score: median (IQR)  *Higher scores denote greater anxiety* | 7.0 (4.0, 8.0) | 7.0 (4.0, 11.0) | 5.0 (2.0, 7.0) | 4.5 (3.0, 6.5)^a^ | 6.0 (3.0, 8.0) | 5.0 (4.0, 10.0) ^a^ |
|  |  |  |  |  |  |  |
| CES total score: median (IQR) | 12.0 (10.0, 13.0) | 12.0 (10.0, 13.0) | 10.0 (9.0, 11.0) | 10.5 (9.5, 11.0) ^a^ | 11.0 (9.0, 13.0) | 11.0 (10.0, 13.0) ^a^ |
| *Lower scores denote better carer quality of life* | |  |  |  |  |  |
| PHQ-9 total score: median (IQR) | 7.0 (3.0, 10.0) | 9.0 (5.0, 11.0) | 4.0 (3.0, 8.0) | 5.0 (3.0, 12.0) | 6.0 (3.0, 9.0) | 7.5 (3.5, 11.5) |
| *Higher scores denote greater depressive symptoms* | |  |  |  |  |  |
| ICECAP-A tariff score: median (IQR) | 0.66 (0.44, 0.92) | 0.64 (0.44, 0.83) | 0.78 (0.66, 0.89) | 0.75 (0.64, 0.87) ^a^ | 0.76 (0.61, 0.89) | 0.73 (0.57, 0.83) ^a^ |
| *Tariff closer to 1 reflect better quality of life ^b^* | |  |  |  |  |  |
| *About the person with ALS* |  |  |  |  |  |  |
| MIND-B^c^: median (IQR)  Disinhibition (transformed %)  Apathy (transformed %)  Stereotypical behaviour (transformed %)  Total raw score (max 36) | 78.1 (56.3, 87.5)  66.7 (50.0, 83.3)  68.8 (37.5, 87.5)  26.5 (17.0, 29.0) | 68.8 (37.5, 81.3)  50.0 (33.3, 66.7)  50.0 (37.5, 75.0)  23.0 (15.0, 24.0) | 81.3 (75.0, 93.8)  83.3 (50.0, 91.7)  87.5 (62.5, 100.0)  31.0 (26.0, 34.0) | 87.5 (75.0, 93.8)  83.3 (50.0, 91.7)  87.5 (62.5, 100.0)  30.0 (26.0, 34.0) | 81.3 (68.8, 93.8)  75.0 (50.0, 91.7)  75.0 (50.0, 100.0)  28.0 (23.0, 33.0) | 81.3 (59.4, 93.8)  58.3 (33.3, 83.3)  68.8 (43.8, 93.8)  25.0 (19.0, 31.0) |
| ALSFRS-R total score: median (IQR) | 31.0 (26.0, 35.0) | 27.0 (16.0, 32.0) | 26.0 (18.0, 35.0) | 23.0 (17.0, 34.0) | 27.0 (21.0, 35.0) | 25.0 (16.5, 33.0) |
|  |  |  |  |  |  |  |

**^a^** 1 participant missing (control group, follow-up) due to incomplete data.

**^b^** ICECAP-A to a capability value between “0” and “1,” where no capability=0, to full capability=1.

**^c^** For MiND-B disinhibition, apathy and stereotypical behaviour subscores, higher scores denote more marked behavioural symptoms. Subscores were transformed to percentages to allow for comparison between subscores, as each subscore has a different maximum raw score. For MiND-B total score, lower raw scores reflect more severe behavioural symptoms. Cut off is ≤ 32/36.

**eTable 3**. MiNDToolkit feasibility trial: Analysis results of questionnaire outcomes at follow-up, using a generalised linear model.

|  |  |  | Untransformed | | Transformed | |
| --- | --- | --- | --- | --- | --- | --- |
| Model outcome at follow-up^a^ | **Intervention group**  **Mean (SD)** | **Control group**  **Mean (SD)** | **Adjusted Difference**  **(95% CI^b^)** | **p-value** | **Adjusted Difference**  **(95% CI^b^)** | **p-value** |
| GAD total score (n=23)^c^ | 7.45 (4.68) | 5.50 (4.52) | -0.07  (-1.69, 1.55) | 0.928 | 0.987  (0.671, 1.452) | 0.944 |
| CES total score (n=23) | 11.45 (2.34) | 10.50 (1.93) | 0.20  (-0.60, 1.00) | 0.613 |  |  |
| PHQ-9 total score (n=24)^c^ | 8.82 (5.27) | 7.38 (6.65) | -0.06  (-2.42, 2.30) | 0.956 | 1.11  (0.85, 1.44) | 0.421 |
| ICECAP-A tariff score (n=23)^c^ | 0.62 (0.24) | 0.71 (0.23) | 0.03  (-0.06, 0.11) | 0.507 | 1.13  (0.84, 1.51) | 0.393 |
|  |  |  |  |  |  |  |

^a^Generalised Linear model used, adjusted for baseline value of outcome variable where available, and treatment group.

^b^95% Confidence Interval for parameter estimates.

^c^Transformed models use a log transformation of the outcome. However, the PHQ-9, MIND-B apathy and ALSFRS models also use a reflection of the outcome before the log transformation, so the interpretation of the direction of score is reversed. The adjusted difference given for the transformed models is the geometric mean ratio (converted back to the original scale).
